# Gut Microbiota in NSAID Enteropathy: Current Evidence and Future Perspectives for Therapeutic Strategies

**DOI:** 10.3390/ph19071045

**Published:** 2026-07-07

**Authors:** Stefania Piccirelli, Brigida Barberio, Enrico Tettoni, Carla Treppiccione, Edoardo Pezzuto, Elisa Tabbone, Daniele Salvi, Luisa Bertin, Viviana Gerardi, Paola Cesaro, Edoardo Vincenzo Savarino

**Affiliations:** 1 Department of Gastroenterology and Endoscopy, Fondazione Poliambulanza Istituto Ospedaliero, 25124 Brescia, Italy; stefania.piccirelli@poliambulanza.it (S.P.); enrico.tettoni@poliambulanza.it (E.T.); carla.treppiccione@poliambulanza.it (C.T.); edoardo.pezzuto@poliambulanza.it (E.P.); elisa.tabbone@poliambulanza.it (E.T.); daniele.salvi@poliambulanza.it (D.S.); viviana.gerardi@poliambulanza.it (V.G.); paola.cesaro@poliambulanza.it (P.C.); 2 Gastroenterology Unit, Department of Surgery, Oncology and Gastroenterology (DISCOG), University of Padova-Azienda Ospedaliera di Padova, 35128 Padova, Italy; luluisa92@gmail.com (L.B.); edoardo.savarino@unipd.it (E.V.S.); 3 Department of Translational Medicine and Surgery, Cattolica del Sacro Cuore University, 00168 Rome, Italy; 4 Gastroenterology Unit, Department of Surgery, Oncology, and Gastroenterology, University of Padova, 35128 Padova, Italy

**Keywords:** NSAIDs toxicity, enteropathy, small bowel disorders, gut microbiota, microbial modulation, therapeutic algorithm

## Abstract

Non-steroidal anti-inflammatory drugs (NSAIDs) are widely prescribed worldwide for their analgesic, antipyretic, and anti-inflammatory properties. However, their long-term use is associated with substantial gastrointestinal (GI) toxicity. Although upper GI injury has traditionally received greater attention, NSAID-induced enteropathy is now increasingly recognized as a common yet underdiagnosed condition. Advances in small-bowel imaging, particularly capsule endoscopy, have demonstrated that mucosal injury of the small intestine occurs in up to 70–80% of chronic NSAID users and may also develop after short-term exposure in otherwise healthy individuals, often without overt clinical symptoms. When symptoms do occur, NSAID-induced enteropathy is characterized by non-specific clinical manifestations and may lead to severe complications in approximately 1% of cases. The pathogenesis of NSAID-induced enteropathy is multifactorial and more complex than that underlying upper GI damage. It involves prostaglandin depletion, increased intestinal permeability, bile acid-mediated toxicity, enterohepatic recirculation of NSAIDs, and, importantly, interactions with the gut microbiota. Concomitant therapies, particularly proton pump inhibitors, may further aggravate small-bowel injury by promoting intestinal dysbiosis. Growing evidence supports a relevant contributory role for the gut microbiota as both a mediator and a modulator of NSAID-induced toxicity, affecting epithelial barrier function, oxidative stress, immune responses, and bile acid metabolism. This review provides an overview of current knowledge of NSAID-induced enteropathy, with a particular emphasis on the microbiota-driven mechanisms underlying mucosal injury. By integrating emerging microbiota-targeted therapeutic approaches, we propose a management algorithm that may help modify disease progression in a condition that remains frequently overlooked in clinical practice.

## 1. Introduction

Non-steroidal anti-inflammatory drugs (NSAIDs) represent one of the most frequently consumed medications globally due to their anti-inflammatory, antipyretic, and analgesic properties [1]. Nevertheless, prolonged exposure to NSAIDs is associated with clinically relevant adverse effects, including injury to the intestinal mucosa throughout the gastrointestinal (GI) tract. While damage to the upper GI tract has classically been linked to prostaglandin inhibition and acid-dependent mechanisms, the pathogenesis of NSAID-induced enteropathy is considerably more complex, involving multifactorial interactions among the intestinal barrier, bile acids, enterohepatic drug circulation, and the gut microbiota [2,3,4]. Technological advances in high-throughput sequencing, metabolomic profiling, and gnotobiotic animal models have substantially refined our understanding of these mechanisms. The intestinal microbiota is now recognized as a key determinant of NSAID-related toxicity, acting both as a mediator and a regulator of mucosal injury by modulating epithelial permeability, redox balance, bile acid composition, and mucosal immune activity [5,6,7]. Growing attention is given to widely prescribed concomitant therapies, such as proton pump inhibitors, which may potentiate small-intestinal damage by promoting inflammatory microbial shifts and altering bile solubility [8]. Despite this growing knowledge, there are still no established clinical strategies to prevent or attenuate NSAID enteropathy beyond discontinuation or substitution of the offending drug.

This review provides a comprehensive overview of the most recent evidence concerning the main features of NSAID-induced enteropathy, its pathophysiology, and the molecular mechanisms by which gut microbiota contribute to NSAID-induced enteropathy. We focus on how the intestinal microbiota and/or its modulation can function as a double-edged sword, exerting either protective and deleterious effects on the small-intestinal mucosa and acting as friend or foe. Unlike previous reviews, this manuscript specifically focuses on microbiota-targeted therapeutic strategies and their translational implications in NSAID-induced enteropathy, integrating both mechanistic insights and emerging clinical evidence. We reviewed the preclinical and clinical evidence of the emerging microbiota-targeted therapeutic approaches, such as antibiotics, probiotics, prebiotics, β-glucuronidase inhibitors, and fecal microbiota transplantation (FMT), and how targeted modulation of the microbiota may alter the natural history of NSAID-induced enteropathy, a condition that is frequently underrecognized in clinical practice.

## 2. NSAID-Induced Gastrointestinal Injury: Heterogeneity in Toxicity and Presentation

Non-steroidal anti-inflammatory drugs (NSAIDs) constitute one of the most commonly prescribed and consumed drug classes worldwide [1]. In 2019, the global market for NSAIDs was estimated at USD 15.58 billion and is expected to expand to approximately USD 24.35 billion by 2027 [9]. However, their widespread use is accompanied by a well-documented spectrum of adverse effects, including renal dysfunction, hepatotoxicity, and an increased risk of major cardiovascular events, in addition to gastrointestinal (GI) injury [10,11]. Data from the EVIDENCE study demonstrated an overall incidence of GI adverse events of 19 per 100 person-years, with complications occurring far more frequently in the upper GI tract than in the lower tract (12% versus 1%) [12]. Among NSAID-related GI complications, peptic ulcer disease has historically been regarded as the most clinically relevant. Prolonged NSAID exposure is estimated to contribute to approximately 36% of all peptic ulcer cases, while symptomatic upper GI ulceration develops annually in about 3–4.5% of NSAID users. Of these patients, nearly 1.5% experience serious complications, including gastrointestinal bleeding, perforation, or obstruction [13,14,15].

In contrast, injury to the small intestine associated with NSAID use has long been considered less prevalent, a perception largely attributable to past technical limitations in visualizing and assessing the small-bowel mucosa. With the advent of capsule endoscopy in the early 2000s, multiple studies have consistently demonstrated a substantial risk of small-bowel injury associated with NSAID use. Mucosal damage was observed in up to 75% of healthy volunteers following short-term oral NSAID exposure [16,17,18]. A high prevalence of small-bowel injury has been reported among chronic NSAID users: in particular, mucosal injury was identified in 71% of patients with arthritis receiving NSAIDs for more than three months, compared with only 10% of non-users [19]. Similarly, mucosal breaks were detected in 81% of long-term NSAID users with rheumatoid arthritis versus 33% of controls, consistently demonstrating significant small-bowel toxicity [20]. NSAID-induced enteropathy is often clinically silent, with most patients remaining asymptomatic despite significant mucosal injury [16,21]. In patients presenting with GI symptoms, small-bowel mucosal injury is detected in approximately 20% of cases. In these patients, symptoms are typically non-specific and may include iron-deficiency anemia due to occult GI bleeding, hypoalbuminemia from protein-losing enteropathy, and, less commonly, abdominal pain, diarrhea, or bloating [22,23,24]. Approximately 1% of patients develop severe complications from NSAID-induced enteropathy, such as overt GI bleeding (melena and/or hematochezia), small-bowel strictures (diaphragm disease), obstruction, and/or perforation [18,25].

Diagnosis of NSAID-induced enteropathy relies on a combination of clinical suspicion including medication history, laboratory markers (i.e., fecal calprotectin), and direct visualization of small-bowel mucosa through video capsule endoscopy (VCE) or device-assisted enteroscopy (DAE). If clinical presentation is unremarkable, medication history is essential. Increase in fecal calprotectin may also support clinical suspicion; however, it does not reliably correlate with the presence or severity of small-bowel lesions [17,18,26]. Thus, no biomarker currently replaces direct visualization through endoscopy for proper diagnosis. Endoscopic evaluation by VCE or DAE can detect a large spectrum of lesions, which vary from reddened folds and red spots, mucosal breaks (erosions and ulcer being the most common), and diaphragm-like strictures [20,25]. Multiple lesions are frequent, and their distribution appears influenced by treatment duration and patient history [27]. In patients with suspected NSAID-induced enteropathy, radiologic imaging, including computed tomography (CT) and magnetic resonance (MR) enterography, has limited sensitivity for detecting small or subtle mucosal lesions. Thus, CT/MR enterography is primarily indicated for assessing intestinal patency and excluding structural complications such as diaphragmatic strictures, deep ulcers, and areas of luminal narrowing [28,29].

The histopathological features of NSAID-induced enteropathy are heterogeneous and often non-specific, though several patterns are consistently observed. Typical findings include focal active enteritis, increased neutrophilic or mixed inflammatory infiltrates, and, in chronic cases, architectural disarray without true distortion. Severe cases may exhibit villous atrophy, epithelial apoptosis, or necrotizing enteritis [30,31]. Diaphragm-like strictures, concentric, thin, web-like fibrotic rings caused by submucosal fibrosis and muscularis mucosae disruption secondary to chronic ulceration, are pathognomonic but rarely captured in routine biopsies [30,32]. Additional findings reported in diaphragm disease include eosinophilic enteritis, inflammatory fibroid polyps, enteritis cystica profunda, and neuromuscular or vascular hamartoma-like changes [33].

Differentiation from Crohn’s disease can be challenging due to overlapping histologic features. Crohn’s disease is typically characterized by transmural inflammation, deep linear or fissuring ulcers, segmental involvement, architectural distortion, and non-caseating epithelioid granulomas (seen in <20% of cases) [34]. Paneth cell hyperplasia and pseudopyloric or pseudofoveolar metaplasia are more common in Crohn’s disease, but they may also occur in NSAID-induced ileitis. To note, pseudopyloric metaplasia has been reported in 23% of Crohn’s cases versus 13% of NSAID-induced cases, whereas pseudofoveolar metaplasia appears more specific for Crohn’s disease [35]. Finally, persistent or progressive mucosal injury despite NSAID withdrawal supports a diagnosis of Crohn’s disease over drug-induced enteropathy.

## 3. NSAID-Induced Enteropathy: Pathophysiology

NSAIDs increase the risk of GI adverse events through inhibition of the prostaglandin–endoperoxide pathway mediated by cyclooxygenase (COX) enzymes [36]. Two main COX isoforms have been identified: COX-1 is constitutively expressed and plays a pivotal role in preserving gastric mucosal integrity, whereas COX-2 is an inducible enzyme upregulated in response to inflammatory cytokines. Based on their chemical structure and selectivity to inhibit COX-1 and COX-2 isoforms, NSAIDs are classified into non-selective (i.e., diclofenac, Ibuprofen, indomethacin, Ketoprofen, Meloxicam, Naproxen, acetylsalicylic acid) and selective (i.e., Celecoxib) NSAIDs. While the therapeutic anti-inflammatory effects of NSAIDs are largely attributable to COX-2 inhibition, GI toxicity predominantly results from COX-1 inhibition, leading to reduced prostaglandin synthesis and subsequent impairment of mucosal defense mechanisms [37,38]. Hence, non-selective NSAIDs and COX-2-selective inhibitors differ substantially in their mechanisms of action and GI toxicity profiles. Non-selective NSAIDs inhibit both COX-1 and COX-2 enzymes and are associated with both upper and lower GI tract injury, including enteropathy and colopathy, with a higher incidence of mild-to-moderate GI events [39,40]. Conversely, COX-2-selective inhibitors, sparing the COX-1 pathway, maintain gastric mucosal protection, resulting in a significantly lower risk of upper GI complications such as ulcers and bleeding, as demonstrated in randomized trials and meta-analyses [39,41]. Also in the small bowel, selective COX-2 inhibitors appear to induce fewer injuries, as confirmed by randomized, double-blind clinical data [17,26,42,43,44]. However, observational studies suggest that the prevalence of small-bowel lesions among chronic COX-2-inhibitor users may approximate that of long-term users of non-selective NSAIDs, implying that the protective advantage may diminish with prolonged exposure [45,46,47]. Acetylsalicylic acid (ASA), known as aspirin, has been traditionally considered safe for the small intestine because of its rapid absorption in the upper GI tract, limited topical activity, and lack of enterohepatic circulation. However, it has similarly been linked to small-bowel injury [48]. Across studies, the prevalence of lesions associated with low-dose aspirin is comparable to that observed with non-selective NSAIDs, particularly with enteric-coated formulations, which expose the small intestine to higher local drug concentrations [49,50,51,52].

The mechanism underlying NSAID-associated damage markedly differs between the stomach and small intestine. Gastric injury is acid-dependent and can be mitigated using proton pump inhibitors (PPIs), while small-bowel damage arises from a more complex interplay between microbiota, bile, and enterohepatic circulation of certain NSAIDs [10,53]. In fact, PPI use can increase the risk of NSAID-induced small-intestine damage, as acid suppression can modify gut microbiota in ways that promote enteropathy [54]. Hypochlorhydria promotes oral bacterial overgrowth and shifts the intestinal microbiota toward an increase in *Enterobacteriaceae* and *Streptococcaceae* species while decreasing beneficial anaerobes, such as *Bifidobacterium* [54,55]. Mechanistically, altered bile composition and bacterial overgrowth synergize with NSAID-induced barrier dysfunction to aggravate mucosal inflammation. Based on capsule endoscopy studies, PPI co-administration induced a five-fold increase in the risk of severe NSAID-induced enteropathy [56,57].

By reducing prostaglandin production, NSAIDs compromise mucosal defense, which is essential for maintaining blood flow, mucus and fluid secretion, and motility [58,59,60]. In parallel, NSAIDs cause mitochondrial dysfunction through uncoupling oxidative phosphorylation, which in turn leads to epithelial cellular changes such as vacuolization, swelling, and loss of cristae, finally leading to cell death and the release of Damage-Associated Molecular Patterns (DAMPs) [61,62,63]. One of the nuclear proteins released by the disruption of cell membranes and mitochondria is the high-mobility group box 1 (HMGB1) protein. Extracellular HMGB1 acts as a DAMP, triggering severe inflammation by activating receptors like Toll-Like Receptor (TLR) 2/4 and RAGE (receptor for advanced glycation end products), which promote cytokine release (TNF-α, etc.) and activate pathways like NF-κB, thus worsening NSAID-induced injury [64]. The NLRP3 inflammasome also contributes to damage by converting pro-IL-1β to active IL-1β, a central mediator of NSAID-induced intestinal injury [65]. Oxidative stress and endoplasmic reticulum (ER) stress further exacerbate mucosal damage: inhibiting the mitochondrial complex I, NSAIDs increase ROS production and DNA damage, while ER stress promotes NLRP3 activation and induces epithelial cell death, weakening barrier integrity [66,67,68].

### 3.1. Microbial Shift Induced by NSAID

**Figure 1:**
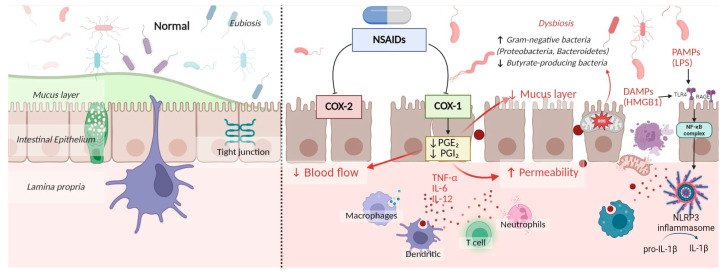
Pathophysiology of NSAID-induced enteropathy. Created in BioRender. (2026) https://BioRender.com/1vemswz (accessed on 15 November 2025).

The oxidative stress produced by NSAID-induced mucosal injury acts as a key driver for the pathological shift in gut microbiota. Oxygen availability plays a fundamental role in shaping intestinal microbial ecology; even modest increases in luminal oxygen levels can favor bacteria with greater tolerance to oxidative conditions, thereby causing dysbiosis [69]. Under conditions of increased luminal oxygen, such as oxidative stress, obligate anaerobes, particularly butyrate-producing bacteria, are selectively depleted. This depletion reflects their pronounced oxygen sensitivity due to the intrinsic vulnerability that catalytic sites of key anaerobic metabolic enzymes have to molecular oxygen and ROS [70]. In parallel, reduced butyrate availability leads to reprogramming of enterocytes and colonocyte metabolism by reducing mitochondrial β-oxidation and epithelial oxygen consumption, perpetuating an environment that favors facultative anaerobic bacteria, reinforcing dysbiosis [71]. As a consequence of this altered niche, NSAIDs induce a pathological shift in the gut microbiota towards pro-inflammatory *Gram-negative* bacteria, such as *Proteobacteria* and *Bacteroidetes* [72,73]. *Proteobacteria*, as facultative anaerobes, adapt their metabolic state based on oxygen availability: they exploit oxygen as a terminal electron acceptor to sustain aerobic respiration, but switch to anaerobic respiration under low-oxygen conditions by using inflammation-derived electron acceptors such as nitrate [74]. Similarly, although *Bacteroidetes* are obligate anaerobes, they exhibit a higher oxygen tolerance compared with butyrate-producing *Firmicutes*. This resilience has been attributed to more robust antioxidant systems, including catalase and superoxide dismutases, as well as to intrinsic bile resistance, an important feature in NSAID enteropathy since exposure to bile acids is usually increased [75,76]. The enhanced resilience of *Gram-negative* bacteria is further supported by their structural characteristics; unlike *Gram-positive* bacteria, *Gram-negative* bacteria have a more complex cell envelope composed of an inner membrane, an outer membrane, and a thin peptidoglycan layer within the periplasmic space. This architecture provides a physical barrier that limits the penetration of reactive oxygen species and other toxic molecules [77]. Consistently, a recent in vitro study demonstrated that oxidative stress reduced butyrate-producing taxa, while facultative aerobics remained largely unaffected and *Bacteroides* exhibited high resilience [75]. (Figure 1).

Beyond compositional shifts, 16S rRNA gene sequencing analyses in murine models demonstrated that indomethacin administration also induces functional changes in microbial metabolic pathways, particularly those related to ATP, heme biosynthesis, and the pentose phosphate pathway [78]. Interestingly, these changes may also confer protection against further injury. As shown by Xiao et al., fecal microbiota transplantation (FMT) from indomethacin-treated mice to naïve recipients reduced susceptibility to subsequent indomethacin injury, suggesting that the post-treatment microbial community had acquired a protective phenotype [78]. However, in confirmation of microbiota resilience, available human studies indicate that NSAID-induced perturbations of intestinal permeability and gut microbiota are reversible after drug withdrawal. As shown in a controlled human study, indomethacin administration was associated with increased permeability and reduced microbial diversity both in fecal samples and duodenal aspirates, as assessed through 16S rRNA gene sequencing. Notably, these alterations returned to baseline within 4–6 weeks after drug discontinuation, with no evidence of persistent or epigenetic alterations [79]. Certainly, as demonstrated since the late 70s, indomethacin fails to induce any significant injury in germ-free mice, whereas colonization with conventional microbiota restores lesion formation [72]. In this scenario, it can be supposed that while a normal commensal community is required to initiate mucosal damage, certain adaptive shifts following exposure to NSAIDs may confer protection against further injury [78]. Moreover, it can be supposed that a long duration of NSAID use is necessary to consistently modify microbial ecology and overcome its resilience.

### 3.2. The Pathogenic Role of Gut Microbiota

The cascade that leads to intestinal damage weaves together the resident gut microbiota, its metabolites and Pathogen-Associated Molecular Patterns (PAMPs). A key event in this process is the increased luminal and systemic exposure to lipopolysaccharide (LPS), a structural component of *Gram-negative* bacteria that acts as a potent PAMP and represents a major driver of microbiota-dependent mucosal injury. Bacterial LPS binds TLR4 on intestinal epithelial and immune cells, activating the MyD88-dependent NF-κB signaling pathway and promoting the release of pro-inflammatory cytokines, including TNF-α, IL-6, and IL-12, together with leukocyte recruitment, ultimately leading to mucosal damage [80,81,82,83,84,85]. Consistent with this mechanism, experimental models have shown that NSAID-induced enteropathy is markedly attenuated in TLR4-deficient mice, confirming the central role of the TLR4–MyD88–NF-κB axis in mucosal inflammation [86].

Additional microbiota-dependent DAMPs contribute to inflammation amplification. Among these, high-mobility group box 1 (HMGB1) is a critical mediator. LPS is a strong experimental inducer of HMGB1 release, which in turn sustains inflammatory signaling through TLR4- and RAGE-dependent NF-κB activation [87,88]. Recent studies have demonstrated that germ-free models colonized with enteric pathogens exhibit significantly increased HMGB1 levels and that pharmacological blockade of HMGB1 and its downstream signaling pathways (including HMGB1/RAGE/NF-κB) reduces NSAID-induced intestinal injury [64,89]. Moreover, HMGB1 inhibition also attenuates LPS-induced mucosal damage through a TLR4-dependent mechanism [90].

Bile acid (BA) metabolism represents another major microbiota-dependent factor in NSAID enteropathy [91]. Gut bacteria such as *Bacteroides*, *Clostridium perfringens*, *Listeria monocytogenes*, *Lactobacillus*, and *Bifidobacterium* convert primary BAs into secondary BAs, a process that can exacerbate intestinal inflammation [92,93]. Secondary BAs, including ursodeoxycholic acid and particularly deoxycholic acid, have been shown to worsen NSAID-induced injury by inducing IL-8 production and activating NF-κB signaling [94,95]. Conversely, germ-free mice, which lack secondary BAs, display reduced susceptibility to NSAID-induced intestinal injury [96]. Enterohepatic circulation provides an additional mechanism by which the gut microbiota further contributes to drug toxicity. Many NSAIDs undergo hepatic glucuronidation, forming acyl-glucuronide conjugates that are excreted into bile and delivered to the intestinal lumen. Here, bacterial β-glucuronidases hydrolyze these conjugates, regenerating the parent drug and thereby re-exposing the intestinal mucosa to toxic aglycones that can be reabsorbed, thus exerting direct topical injury [97,98,99]. In parallel, *Gram-negative* bacteria and their LPS components further amplify mucosal inflammation through TLR4 activation [90]. In addition, NSAIDs competitively bind BAs and phosphatidylcholine, forming cytotoxic mixed micelles that disrupt phospholipid membranes and further exacerbate mucosal injury. Despite these findings in preclinical studies, there is no clinical evidence that UDCA-NSAID association could result in any worsening of small-bowel damage. Hence, UDCA use in patients taking NSAIDs should follow the common indications [100,101].

Gut microbiota-derived metabolites play a fundamental role in maintaining epithelial integrity and immune homeostasis. These include short-chain fatty acids (SCFAs), BAs, branched-chain amino acids, trimethylamine N-oxide, tryptophan derivatives, and indoles, which collectively support epithelial energy metabolism and barrier function [102]. SCFAs play a particularly important role in enhancing barrier integrity by activating NLRP3-dependent IL-18 release [103,104]. Hence, under dysbiotic conditions, their depletion may exacerbate NSAID-induced injury, especially in older adults with long-term NSAID use who exhibit decreased levels of SCFAs such as isobutyrate, isovalerate, and L-lactate [103]. Under dysbiotic conditions, other metabolites can promote intestinal barrier dysfunction and inflammation, such as serotonin, a neurotransmitter primarily produced by enterochromaffin cells in the GI tract. Serotonin (5-HT) production, stimulated by SCFAs and secondary BAs, was increased in NSAID-treated mice [105]. By activating 5HT3 receptors, 5HT promotes intestinal hypermotility, a pathogenic event related to NSAID enteropathy, which increases mechanical stress on the mucosa and disrupts the protective mucus layer, facilitating bacterial penetration into the mucosa [106,107,108].

## 4. Strategies of Microbiota Modulation to Prevent NSAID-Induced Enteropathy

To prevent upper GI NSAID-related toxicity, national and international food and drug agencies recommend co-prescribing PPIs in patients necessitating NSAID use and contemporarily presenting with increased risk for GI injury (i.e., concomitant use of aspirin, anticoagulants, and/or previous history of GI ulcer) [109,110]. On the contrary, to prevent small-bowel toxicity, no intervention with proven efficacy is recommended in clinical practice to prevent NSAID-induced small-bowel injury. Paradoxically, PPIs have been demonstrated to worsen NSAID-induced damage, negatively influencing gut microbiota, as discussed in the above section. These conflicting findings should be interpreted within the overall clinical context; as PPIs remain an established strategy to prevent upper GI NSAID-related complications, their use should be guided by an individualized risk–benefit assessment rather than routinely discouraged [53]. Environmental factors, including antibiotic exposure, smoking, and stress, also modulate the microbiota and potentially influence NSAID toxicity. Given the relevant role of gut microbiota in the pathogenesis of NSAID-induced enteropathy, growing attention has been directed to therapeutic strategies that can modulate intestinal microbiota. Currently, approaches under investigation include non-absorbable antibiotics, a variety of probiotics, dietary compounds, and microbiota-derived metabolites. Although heterogenous, these interventions share the common aim of mitigating mucosal injury by restoring microbial balance, enhancing epithelial barrier function, and attenuating intestinal inflammation. Most of the current evidence comes from basic science; however, endoscopic and capsule-based studies have translated these experimental findings into clinical relevance (Table 1).

### 4.1. Antibiotics

Antibiotic modulation of the intestinal microbiota represents one of the earliest and most validated strategies for preventing NSAID-induced enteropathy. Broad-spectrum antibiotics such as neomycin and metronidazole were shown decades ago to attenuate indomethacin-induced intestinal injury in rodents [6]. In 2007, Watanabe et al. demonstrated that antibiotics targeting *Gram-negative* bacteria, such as Ampicillin or Aztreonam, reduce indomethacin-induced small-intestine damage. On the contrary, antibiotics targeting *Gram-positive* bacteria, such as Vancomycin, do not confer protection [80]. More recently, rifaximin—a non-absorbable, eubiotic antibiotic—was demonstrated to reduce mucosal injury when co-administered with diclofenac by decreasing pro-inflammatory bacterial phyla and by suppressing pro-inflammatory signaling via TLR and inflammasome pathways. In the diclofenac–enteropathy rat model, rifaximin co-administration normalized microbiota composition (restoring beneficial taxa such as *Lactobacilli*, typically depleted after NSAID exposure), increased occludin expression, and reduced mucosal cytokine production and NLRP3 activation, thus reducing oxidative stress in the intestinal mucosa [111]. Remarkably, these modulating effects were not observed when rifaximin was administered in rats without concomitant NSAID use, suggesting that its impact emerges specifically in the context of NSAID-induced perturbations. These preclinical findings were corroborated in a randomized, placebo-controlled clinical trial assessing NSAID-induced injury by CE. In 60 healthy volunteers receiving diclofenac and Omeprazole, a 14-day regimen of rifaximin (400 mg twice daily) significantly reduced the incidence of small-bowel mucosal breaks compared to placebo (20% vs. 43%) [112].

These findings suggest that rifaximin may represent a promising strategy for the prevention of NSAID-induced enteropathy, particularly when co-prescribed with PPI. However, the available clinical evidence remains limited and insufficient to support its routine preventive use. Moreover, long-term treatment should be used with caution due to the risk of antibiotic resistance. Future clinical trials are needed to establish its clinical validity and safety before firm recommendations can be made.

### 4.2. Probiotics

Probiotics represent another emerging area of investigation. Most candidate microbes have been evaluated in preclinical models, revealing promising mechanisms of protection that remain to be validated in humans. For example, in a murine indomethacin-induced enteropathy model, oral administration of *Lactobacillus Casei CRL 431* and *Lactobacillus Paracasei CNCM I-1518* attenuated intestinal injury, assessed through macroscopic examination and histological analysis, preserving villus architecture, Paneth and Goblet cell populations, antimicrobial activity, and reducing pro-inflammatory cytokines [113]. Similarly, pretreatment with *Lactobacillus Casei Shirota* protected against indomethacin-induced small-intestinal injury through increased local lactic acid production, which suppresses the LPS/TLR4-driven inflammatory cascade, central to induction of NSAID enteropathy [114]. A comparable protective pattern has been reported for *Saccharomyces Boulardii* CNCM I-745 in a rat model of diclofenac-induced enteropathy [115]. Diclofenac not only altered microbiota in fecal and ileal samples, but also increased fecal butyrate levels, reflecting reduced mucosal absorption secondary to downregulation of the butyrate transporter. Administration of *Saccharomyces Boulardii CNCM I*-745 markedly reduced histological damage, inflammatory markers (myeloperoxidase, TNF, and IL-1 beta), oxidative stress, and microbiota alterations. Notably, only preventive treatment restored butyrate-transporter expression and normalized butyrate levels, highlighting the importance of timing in probiotic-based strategies [115]. *Bifidobacterium Adolescentis*, a commensal species, exerts an anti-inflammatory effect by suppressing TNF-alfa and other cytokines through its cell surface polysaccharide, partly by competing with LPS for CD14 binding to CD14, and inhibiting activation of the TLR4-NFkB pathway [116]. Consistent with these observations, *Bifidobacterium Adolescentis* significantly reduced Naproxen-induced enteropathy in rats, although the precise protective mechanism remains incompletely defined [7].

Although most evidence derives from murine models, human studies have begun to explore the potential protective effects of probiotics against NSAID-induced small-bowel injury. A double-blind study in 75 healthy volunteers receiving acetylsalicylic acid demonstrated that co-administration of *Bifidobacterium breve Bif195* significantly reduced small-bowel damage, as documented by VCE [117]. Mucosal damage was quantified using the Lewis score, which evaluates villous edema, ulcers and stenosis, and by counting red spots [118]. Importantly, this protective effect occurred without altering systemic thromboxane B_2_ levels, indicating no interference with aspirin’s antiplatelet activity. Although the molecular mechanism underlying this protective action remains unclear, one proposed factor is the pilus-associated protein Tad E, which exerts a proliferative effect on host colonic epithelium after oral ingestion of *Bifidobacterium breve Bif195* [117]. A similar protective effect has been observed with *Lactobacillus Gasseri (LG) OLL2716*, in a randomized, double-blind, placebo-controlled trial. In this study, twice-daily consumption of yogurt containing *LG OLL2716* for six weeks significantly reduced aspirin-induced small-bowel injury [119]. Mucosal lesions, including ulcers and bleeding, were assessed by VCE before and after the intervention, demonstrating a clear protective effect compared with placebo. Evidence of probiotic-mediated protection in humans is further supported by a double-blind study in healthy volunteers, in which the probiotic mixture *VSL#3*, administered before and during indomethacin exposure, attenuated NSAID-induced intestinal inflammation [120]. While placebo resulted in a sustained rise in FC over several days, VSL#3 limited this increase to a single day, suggesting a protective effect against indomethacin-induced intestinal inflammation. However, not all probiotics demonstrate similar efficacy. In a randomized, placebo-controlled, double-blind study conducted on healthy volunteers, administration of *Bifidobacterium animalis subsp. lactis 420 (B420)* before and during diclofenac administration failed to prevent an NSAID-associated rise in FC, with increases comparable to those observed in the placebo group [121]. Therefore, despite encouraging results from selected studies, available evidence indicates that the protective effects of probiotics against NSAID-induced enteropathy is strain-specific, highlighting the need for further studies to identify the most effective probiotic strains, treatment regimens, and target populations before clinical use can be recommended.

### 4.3. Dietary Compounds and Microbial Metabolites

Dietary patterns critically modulate the composition and resilience of the intestinal microbiota, thereby influencing susceptibility to NSAID-induced injury. Based on experimental evidence, high-fat Western diets (HFDs) exacerbate enteropathy by increasing *Proteobacteria* and *Desulfovibrionaceae* abundance, enhancing endotoxin load, and upregulating IL-17A-mediated inflammation [122]. Sugimura et al. demonstrated that mice fed with an HFD developed more severe indomethacin-induced intestinal injury than those on standard chow and that IL-17A neutralization or microbiota transplantation from control mice significantly ameliorated the damage [122]. Arron et al. evaluated the synergistic effects of diet and NSAIDs on intestinal anastomotic healing in a murine model: a Western diet combined with diclofenac caused dysbiosis, epithelial barrier failure, and anastomotic leakage—an effect not observed with either factor alone. In contrast, high-fiber and plant-based diets enhance microbial diversity, promote SCFA production, particularly butyrate, and are associated with reduced intestinal inflammation and improved barrier function, conferring mucosal protection. Wallace et al. observed that soluble fibers such as pectin and guar gum enhanced SCFA production and reduced jejunal and ileal damage by 60–90% in rats without compromising NSAID anti-inflammatory efficacy [123]. Moreover, diets enriched in polyphenols, resistant starch, and omega-3 fatty acids have been proposed to enhance microbial-derived butyrate synthesis, further stabilizing epithelial tight junctions [124]. Among polyphenols, resveratrol has attracted growing interest for its potential protective effects. In a rat model of NSAID-induced intestinal injury under hypoxic conditions, resveratrol administration attenuated mucosal damage, reduced pro-inflammatory cytokines (IL-1β, TNF-α), increased anti-inflammatory IL-10, and restored tight-junction proteins (ZO-1, Occludin) [125]. These effects were accompanied by downregulation of the TLR4/NF-κB/IκB signaling pathway and significant shifts in gut microbiota, including increased abundance of Clostridium following treatment [125]. Mechanistically, the literature supports that resveratrol can modulate gut microbiota, promote beneficial bacterial populations, and enhance intestinal barrier integrity, features highly relevant to the pathogenesis of NSAID enteropathy [126].

Human studies on dietary interventions in NSAID enteropathy remain lacking. A recent study on healthy volunteers evaluated a prebiotic strategy to increase the abundance of *Faecalibacterium Prausnitzii*, a *Gram-positive* commensal butyrate-producing bacterium that plays a protective role in NSAID enteropathy by exerting anti-inflammatory effects, supporting gut homeostasis and intestinal barrier integrity [127]. The authors demonstrated that oral supplementation with maltobionic acid and lactobionic acid can increase *Faecalibacterium* abundance, an effect apparently mediated by Parabacteroides species, which convert the gluconic acid component of these oligosaccharides into glucuronic acid, a substrate utilized by *Faecalibacterium Prausnitzii*, thereby promoting its growth [128].

Microbiota-derived metabolites have also emerged as promising candidates. A murine study demonstrated that indole, a tryptophan-derived metabolite produced by commensal bacteria (i.e., *Lactobacillus reuteri*), significantly attenuates indomethacin-induced enteropathy. Co-administration of indole with NSAIDs reduced mucosal inflammation, histological damage, and fecal calprotectin levels. In addition, indole prevented NSAID-induced dysbiosis and normalized the pro-inflammatory transcriptional profile of the intestinal mucosa. These findings suggest that microbiota-derived metabolites may play a protective role in the pathogenesis of NSAID enteropathy [86]. All of the abovementioned evidence underscores the multifactorial nature of NSAID enteropathy and the need to consider host dietary background in both experimental and clinical settings [129].

Although dietary and prebiotic interventions show promising microbiota-modulating effects, most of the available evidence derives from preclinical models, and robust clinical data are still lacking.

### 4.4. Other Therapeutic Strategies

Bacterial β-glucuronidase inhibition has emerged as another successful preclinical strategy to decouple NSAID therapeutic efficacy from intestinal toxicity. LoGuidice et al. showed that selective inhibition of this enzyme prevented diclofenac-induced mucosal ulceration in mice, without affecting systemic pharmacodynamics or bacterial viability. By blocking the deconjugation of acyl-glucuronides, these agents disrupt enterohepatic recirculation and prevent luminal accumulation of toxic NSAID metabolites. β-glucuronidase inhibitors thus represent a promising class of microbiota-targeted drugs warranting further clinical evaluation [130]. FMT was also considered as a potential therapeutic strategy due to its capability to restore a healthy microbial ecosystem and mitigate NSAID-induced injury [78]. Wang et al. proposed FMT for refractory enteropathy based on its ability to restore microbial diversity, increase SCFA production, suppress LPS/TLR4 signaling, and modulate immune responses [8].

Although no clinical trials have yet specifically addressed FMT in NSAID enteropathy, its success in other gastrointestinal conditions such as *Clostridioides difficile* infection and inflammatory bowel disease provides a strong rationale for future research [57].

Additional experimental strategies include hydrogen sulfide-releasing NSAIDs [131], prostaglandin analogs (misoprostol, rebamipide), and bile acid sequestrants [123,132]. These agents act by preserving mucosal perfusion or reducing detergent bile cytotoxicity rather than directly altering the microbiota. In this scenario, they may synergize with microbiota-targeted therapies due to the interplay between microbiota, bile acid receptors (FXR, TGR5), and mucosal immune signaling [86,122]. However, no clinical studies have addressed these agents. To mention misoprostol, its efficacy and safety should be tested in a clinical setting due to its abortifacient properties, and its use might be restricted to selected patients.

## 5. Current Limitations and Future Perspectives

Although growing evidence strongly supports the protective role of intestinal microbiota in NSAID-induced enteropathy, most available data still derive from animal models, and translation into clinical practice remains limited. Human studies are few, and only a small number have evaluated small-bowel mucosal injury using endoscopy or VCE. Taken together, these limitations underscore the need for well-designed human clinical trials that integrate microbiota profiling with endoscopic endpoints, clarifying the therapeutic potential of microbiota-directed interventions in NSAID enteropathy. Nevertheless, the recognition that the gut microbiota plays a causal role in NSAID enteropathy reshapes the therapeutic options that are currently applied to treat this condition, limited to NSAID withdrawal and endoscopic/surgical procedures to treat eventual small-bowel stenoses and/or ulcerative lesions. Moreover, new insights into the role of gut microbiota open the doors to several clinical strategies which go beyond the current classical preventive strategies, such as acid suppression (that was demonstrated to even be harmful), and that could be integrated into current clinical practice. This review collects the most recent evidence in this field and introduces a potential pharmacological strategy that is “gut microbiota-centered” to treat NSAID enteropathy. As shown in human studies, particular probiotic strains such as *Bifidobacterium breve Bif195*, *Lactobacillus gasseri OLL2716*, and *VSL#3* have been demonstrated to attenuate small-bowel inflammation in patients using NSAIDs. Indirectly, the same result was reached by prebiotic compounds such as maltobionic acid and lactoionic acid, which were demonstrated to increase the abundance of Faecalibacterium prausnitzii, a notable anti-inflammatory bacterium. Prostaglandin analogs, such as misoprostol and rebamipide, could also play a role in reducing mucosal damage.

**Figure 2:**
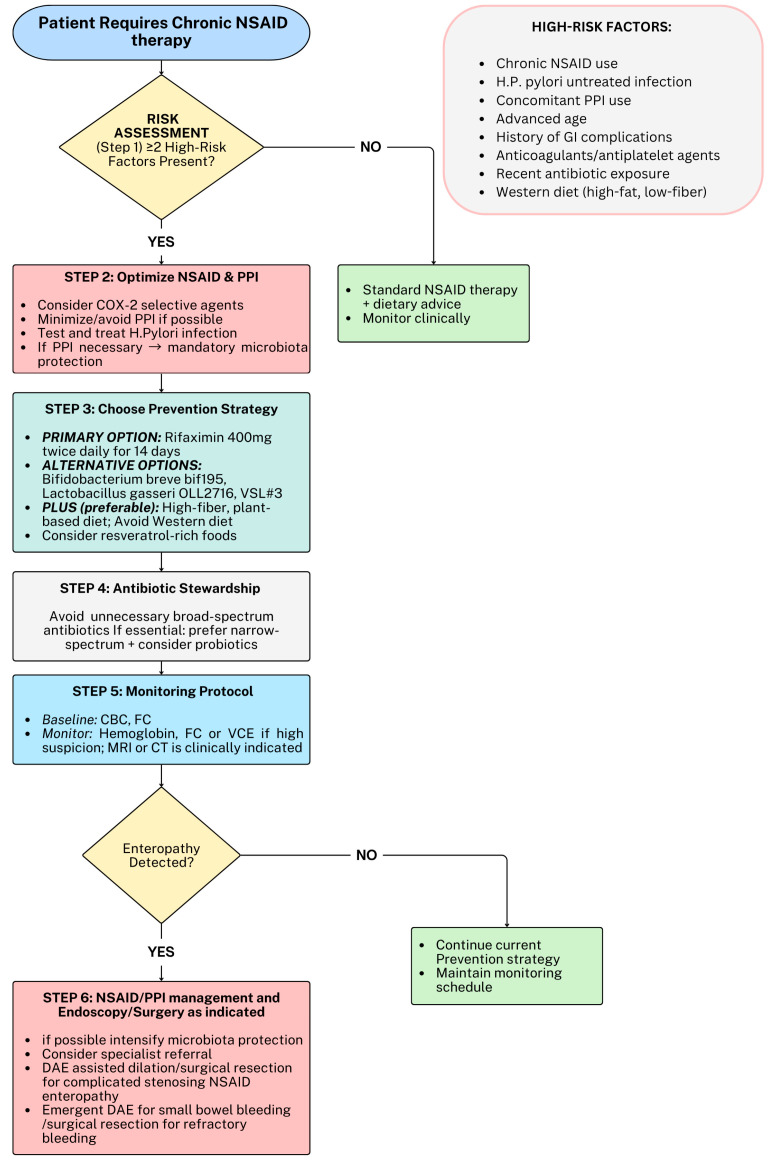
Proposed algorithm for clinical management and prevention of NSAID enteropathy. PPI: Proton pump inhibitor; GI: gastrointestinal; FC: fecal calprotectin; CBC: complete blood count; VCE: videocapsule endoscopy. This framework is intended for conceptual purposes only and does not represent a validated clinical decision-making tool.

First, PPI co-administration, advanced age, and chronic NSAID exposure should be assessed to predict and prevent small-bowel injury: avoidance of unnecessary PPI use in long-term NSAID users without upper GI risk factors could represent an option, as well as considering COX-2-selective agents when appropriate, although their use should be balanced against potential cardiovascular and renal risks, as well as considering that the risk of small-bowel injury is not eliminated [3,56,133,134]. Second, promoting a high-fiber, plant-based diet to sustain SCFA-producing bacteria, as well as avoiding broad-spectrum antibiotic overuse, could help to mitigate mucosal damage [111,122,123]. Third, rifaximin or targeted probiotic supplementation could also be considered in high-risk patients, while FC and hemoglobin levels may help to detect and monitor subclinical enteropathy. However, long-term use of rifaximin should be discouraged in order to prevent antibiotic resistance [8,57,135] A conceptual, expert-opinion framework for the prevention and management of NSAID enteropathy is proposed in Figure 2 and it should not be interpreted as a clinical decision-making tool.

In the near future, multi-omics integration, combining metagenomics, metabolomics, and transcriptomics, will better delineate host–microbiota–drug interactions. Similarly, randomized, placebo-controlled studies evaluating microbiota-targeted interventions (rifaximin, probiotics, β-glucuronidase inhibitors, FMT) using either standardized endoscopic or biochemical outcomes will clarify how to effectively prevent and treat NSAID gastrointestinal toxicity, ultimately improving patient safety.

Literature Search Strategy: This narrative review was conducted through a comprehensive literature search of the PubMed/MEDLINE database aimed at identifying studies investigating the pathophysiology of NSAID-induced enteropathy, with a particular focus on gut microbiota-related mechanisms and microbiota-targeted therapeutic strategies.

The PubMed/MEDLINE database was systematically searched for articles published up to September 2025. The search strategy combined Medical Subject Headings (MeSHs) and free-text terms, including: “non-steroidal anti-inflammatory drugs”, “NSAIDs”, “enteropathy”, “small bowel injury”, “small intestinal damage”, “intestinal permeability”, “gut microbiota”, “microbiome”, “dysbiosis”, “probiotics”, “prebiotics”, “antibiotics”, “fecal microbiota transplantation”, and “β-glucuronidase”. Boolean operators (“AND”, “OR”) were used to optimize the search.

Eligible studies included both preclinical (in vitro and animal models) and clinical investigations (randomized controlled trials, cohort studies, case–control studies, and observational studies) addressing epidemiology, pathophysiology, host–microbiota interactions, and microbiota-targeted interventions in NSAID-induced small-bowel injury. Only articles published in English were considered. Additional relevant studies were identified through manual screening of reference lists from selected articles and review papers.

Studies focusing exclusively on upper gastrointestinal toxicity, case reports, conference abstracts, editorials, and non-peer-reviewed literature were excluded. Retrieved records were screened based on title and abstract, followed by full-text evaluation of potentially relevant articles. Priority was given to studies providing mechanistic insights into microbiota-mediated intestinal injury and to clinical studies evaluating diagnostic, preventive, or therapeutic strategies for NSAID-induced enteropathy.

## Figures and Tables

**Table 1:** Microbiota-targeted interventions for prevention and treatment of NSAID-induced enteropathy: mechanisms, evidence, and translational applicability.

Category	Intervention	Proposed Microbiota-Related Mechanism	Preclinical Evidence	Human Evidence	Main Findings	Current Translational Status
Antibiotics	Neomycin	Reduction of bacterial load	Rat (indomethacin)	No	Reduced intestinal injury	Experimental
	Metronidazole	Anaerobic modulation	Rat (indomethacin)	No	Attenuation of mucosal inflammation	Experimental
	Ampicillin	Reduction of Gram-negative bacteria	Rat (indomethacin)	No	Reduced small bowel injury	Experimental
	Aztreonam	Selective Gram-negative depletion	Rat	No	Protection against mucosal injury	Experimental
	Vancomycin	Gram-positive depletion	Rat	No	No protective effect observed	Experimental
	Rifaximin	Microbiota remodeling and reduced inflammatory signaling	Rat (diclofenac)	Healthy volunteers (diclofenac + omeprazole)	Reduced mucosal lesions and partial microbiota normalization	Limited clinical evidence
Probiotics	*Lactobacillus casei* CRL431	Barrier protection and cytokine modulation	Rodent	No	Preserved villus structure and reduced inflammation	Experimental
	*Lactobacillus paracasei* CNCM I-1518	Maintenance of mucosal integrity	Rodent	No	Reduced enteropathy severity	Experimental
	*Lactobacillus casei* Shirota	Increased lactic acid production; reduced TLR4 activation	Rodent	No	Attenuated inflammatory signaling	Experimental
	*Saccharomyces boulardii* CNCM I-745	Anti-inflammatory and antioxidant effects	Rat	No	Reduced oxidative injury and restored butyrate transport	Experimental
	*Bifidobacterium adolescentis*	TLR4/NF-κB modulation	Rat	No	Reduced inflammatory injury	Experimental
	*Bifidobacterium breve* Bif195	Enhancement of mucosal homeostasis	No	Healthy volunteers	Reduced capsule endoscopy injury scores	Preliminary human evidence
	*Lactobacillus gasseri* OLL2716	Barrier support and anti-inflammatory effects	No	Healthy volunteers	Reduced ulcers and bleeding	Preliminary human evidence
	VSL#3	Broad microbial modulation	No	Healthy volunteers	Reduced inflammatory response	Preliminary human evidence
	*Bifidobacterium animalis* subsp. *lactis* B420	Microbiota modulation	No	Healthy volunteers	No significant benefit	Negative clinical study
Dietary/Prebiotic strategies	High-fat Western diet	Dysbiosis and endotoxin amplification	Mouse	No	Exacerbated enteropathy	Mechanistic evidence only
	High-fiber/plant-based diets	Increased SCFA production	Rat	Limited	Improved barrier integrity	Hypothesis-generating
	Soluble fibers (pectin, guar gum)	Enhanced SCFA synthesis	Rat	No	Reduced mucosal injury	Experimental
	Polyphenols/resveratrol	Modulation of microbiota and TLR4/NF-κB	Rat	No	Reduced inflammatory damage	Experimental
	Resistant starch/omega-3 fatty acids	Increased butyrate production	Rat	No	Enhanced epithelial resilience	Experimental
	Maltobionic acid/lactobionic acid	Promotion of *Faecalibacterium prausnitzii*	No	Healthy volunteers	Increased abundance of beneficial taxa	Early clinical evidence
Microbial metabolites	Indole	Anti-inflammatory signaling and microbiota stabilization	Rodent	No	Reduced enteropathy and dysbiosis	Experimental
Other microbiota-oriented strategies	β-glucuronidase inhibitors	Prevention of toxic NSAID recirculation	Rodent	No	Reduced ulceration	Preclinical only
	Fecal microbiota transplantation	Restoration of microbial diversity	Mouse	No	Improved barrier and immune regulation	Research only
	Hydrogen sulfide–releasing NSAIDs	Reduced oxidative injury	Rodent	No	Preserved mucosal perfusion	Experimental
	Misoprostol/rebamipide	Mucosal protection independent of microbiota	Preclinical + limited human	Limited	Reduced intestinal damage	Emerging
	Bile acid sequestrants	Reduced bile-mediated toxicity	Rodent	No	Improved epithelial protection	Experimental

Abbreviations: SCFA, short-chain fatty acids; TLR4, Toll-like receptor 4; NF-κB, nuclear factor kappa B; FMT, fecal microbiota transplantation. Evidence interpretation: “Experimental” = preclinical only; “Preliminary human evidence” = limited human interventional.

## Data Availability

No new data were created or analyzed in this study.
